# BALANCING THE SCALES: ADDRESSING WEALTH DISPARITIES AMONG SEXUAL AND GENDER MINORITY (SGM) OLDER ADULTS

**DOI:** 10.1093/geroni/igae098.0968

**Published:** 2024-12-31

**Authors:** Charles Hoy-Ellis, Karen Fredriksen-Goldsen

**Affiliations:** Goldsen Institute, University of Washington School of Social Work, Seattle, Washington, United States; University of Washington, Seattle, Washington, United States

## Abstract

While the National Institutes of Health, and National Academies of Science, Engineering, and Medicine have recognized LGBTQ+ populations as experiencing significant health disparities, they remain understudied and underserved. Decades of research highlights socioeconomic positionality as a significant driver of health disparities. Our team was the first document extensive health disparities among LGBTQ+ older adults. More recently, we are documenting significant economic disparities, including higher rates of poverty – mediated and moderated by lower rates of marriage and living alone, currently, and historically. It has been less than 10 years since LGBTQ+ people have been afforded the economic privileges and benefits that are inherent to state-sanctioned marriage (more than 1,100 according to the General Accounting Office), which are cumulative over time. Utilizing data from Aging with Pride: National, Health, Aging and Sexuality/Gender Study, the first and largest national longitudinal study of LGBTQ+ older adults (N = 2,450), we identify disparities in wealth and financial resources that function as risk and protective factors predicting economic outcomes among this historically marginalized population. Our findings indicate that despite significantly higher educational achievement compared to their heterosexual age-peers, LGBTQ+ older adults are disadvantaged economically, due in large part to historical sociocultural factors. More research is needed to understand underlying mechanisms of risk and protective factors that influence the unique economic and wealth disparities among LGBTQ+ and other diverse communities to inform the development of targeted interventions to address the unique economic challenges impacting their health and well-being across the life course.

